# A systematic review of patient access to medical records in the acute setting: practicalities, perspectives and ethical consequences

**DOI:** 10.1186/s12910-020-0459-6

**Published:** 2020-03-02

**Authors:** Stephanie N. D’Costa, Isla L. Kuhn, Zoë Fritz

**Affiliations:** 1 Gonville and Caius College, Cambridge University, Trinity Street, Cambridge, CB2 1TA UK; 2 THIS Institute (The Healthcare Improvement Studies Institute), Cambridge University, Clifford Allbutt Building, Cambridge, CB2 0AH UK

## Abstract

**Background:**

Internationally, patient access to notes is increasing. This has been driven by respect for patient autonomy, often recognised as a primary tenet of medical ethics: patients should be able to access their records to be fully engaged with their care. While research has been conducted on the impact of patient access to outpatient and primary care records and to patient portals, there is no such review looking at access to hospital medical records in real time, nor an ethical analysis of the issues involved in such a change in process.

**Methods:**

This study employed a systematic review framework in two stems, to integrate literature identified from two searches: Medline, CINAHL and Scopus databases were conducted, (for (1) hospitalised patients, patient access to records and its effects on communication and trust within the doctor-patient relationship; and (2) patient access to medical records and the ethical implications identified). The qualitative and quantitative results of both searches were integrated and critically analysed.

**Results:**

3954 empirical and 4929 ethical studies were identified; 18 papers representing 16 studies were identified for review (12 empirical and 6 ethical). The review reveals a consensus that our current approach to giving information to patients – almost exclusively verbally – is insufficient; that patient access to notes is a welcome next step for patient-centred care, but that simply allowing full access, without explanation or summary, is also insufficient. Several ethical implications need to be considered: increased information could improve patient trust and knowledge but might transfer an (unwelcome) sense of responsibility to patients; doctors and patients have conflicting views on how much information should be shared and when; sharing written information might increase the already significant disparity in access to health care, and have unforeseen opportunity costs. The impact on medical practice of sharing notes in real time will also need to be evaluated.

**Conclusions:**

The review presents encouraging data to support patient access to medical notes. However, sharing information is a critical part of clinical practice; changing how it is done could have significant empirical and ethical impacts; any changes should be carefully evaluated.

## Background

It is unusual for patients to request access to their medical hospital records, despite their legal right to do so [1]. The U.K. government mandated that patients should be able to readily access their electronic medical record by 2018, a promise which has not been fulfilled, mostly due to logistical difficulties [2]. This mandate was built on respect for patient autonomy as a primary tenet of medical ethics: patients should be able to access their records to be fully engaged with their care. Access to records allows patients to be more informed which may increase opportunities for them to question their care plans and request second opinions.

Internationally, patients are more readily able to access their notes, and there has been evidence of positive outcomes in maternity records [3]; in primary care [4, 5]; for specific diseases, [6, 7] and for specific interventions [8, 9]. A 2003 (Ross and Lin) [10] and 2007 (Ferreira et al) [11] review of the literature in these fields found that patient access was unlikely to cause harm and can improve doctor-patient communication and relations; the latter review also identified the potential for patients to spot and correct mistakes in their records.

More recently, the use of patient ‘portals’ – an electronic route to targeted parts of the medical record – has become more common. Several systematic reviews on the design, use and impact of such portals have been conducted [12–14]. Patients are generally enthusiastic about the possibility of accessibility, and positive or neutral health outcomes were observed. However, it was noted that clinician contact for portal users increased, and, perhaps related to this, disparity of uptake among different ethnic and socioeconomic groups was noted.

While these reviews demonstrate significant bodies of research on the impact of patient access to outpatient and primary care records and to patient portals (see Table 1 for a summary table of the systematic reviews in these domains), there is no such review looking at access to hospital medical records in real time, nor an ethical analysis of the issues involved in such a change in process.

**Table 1:** Summary of systematic reviews conducted on overlapping literatures

Author and Title of paper	Sample Size and setting	Nature of Analysis	Summary of results
Electronic Patient Portals: Evidence on Health Outcomes, Satisfaction, Efficiency and Attitudes. (Goldzweig et al., 2013) [12]	46 included articles: 18 on health outcomes, 7 on efficiency/utilization, 10 on patient characteristics, 19 on attitudes All outpatient setting assessing portals as a way of accessing information. Predominantly US (43/46)	3 systematic literature searches of PubMed and Web of Science spanning different timeframes for the **effects of portals on patient care**. Included reference -mined articles assessed independently by two reviewers.	• **Examined health outcomes for specific diseases** (e.g. effect of intervention on HbA1C levels in diabetics) rather than patient communication and doctor-patient relationship. • **Health outcomes, satisfaction and adherence:** positive or neutral outcomes in intervention patients compared to control patients in RCTs for patient portals for those with chronic conditions; possible confounding factors, as portals used in conjunction with intensive or pharmaceutical-led case management. • **Efficiency or Utilization:** either no difference or increased clinician contact. • **Patient characteristics:** disparity between racial and ethnic groups, literacy or education levels and medical problems in regard to whether the patients were likely to use portals or not, suggesting this is a barrier to accessible portal use.
Inpatient Portals for Hospitalized Patients and Caregivers: A Systematic Review (Kelly et al., 2017) [13]	17 studies All inpatient setting, focusing on design of portals. Predominantly US (15/17)	Systematic literature search of PubMed, Web of Science, CINAL Plus, Cochrane and Scopus for **patient portals, engagement and inpatient care.**	**Examined the design, use and impact of patient portals.** • Portals provided targeted access to information for patients and were varied in their design and content. • Patients generally found portals easy to use and have a positive experience, feeling more engaged and in control; they suggested future portals should have more information, personalised medication and results in real time. • Professional concern over giving information without interpretation and overuse of messaging tools.
Patient engagement in the inpatient setting: a systematic review. (Prey et al., 2014) [14]	17 studies All Inpatient setting focusing on patient engagement. Predominantly US (16/17)	Systematic literature search of PubMed, ACM Digital Library, IEEE Xplore and Cochrane databases for **patient engagement, involvement of health I.T. in an inpatient setting** (English language only)	**Examined what interventions to improve patient access engagement via health information technology were available.** • Five groups were identified: entertainment, general health I.T. delivery, patient-specific information delivery, advanced communication tools and personalized decision report. • Noted limited research on impact on health outcomes and cost-effectiveness.
The effects of promoting patient access to medical records: a review (Ross & Lin, 2003) [10]	30 studies including medical outpatients (14 studies), inpatients (2 studies), obstetric (5 studies) and psychiatric patients (5 studies). Predominantly UK (13/30) and US (13/30)	Systematic literature search of MEDLINE and HealthSTAR searching for the effects of patient access to notes on patient participation and advocacy. Reference mining was also used.	**Examined effects of patient access to records over a period of time on the patient, the doctor-patient relationship and medical practice.** • Patient access to medical records was unlikely to cause harm and generally had modest benefits, especially surrounding doctor-patient communication, seen clearly in three trials in obstetric intervention patients. • Patient satisfaction was generally high, despite some patients finding the records worrisome, upsetting or difficult to understand.
The patient perspective on the effects of medical record accessibility: a systematic review (Vermeir et al., 2017) [15]	12 studies, majority focussing on outpatients [9] and the rest were mixed outpatient and inpatient [3]. Predominantly US (8/12)	Systematic literature search of PubMed, Web of Science, Cinahl and Cochrane Library for effects of communicating medical record information on patient participation and the doctor-patient relationship. The studies were assessed for methodological quality and those scoring average and high ratings were included.	**Examined patient use of and perspectives on medical record accessibility** • Many patient participants were knowledgeable and enthusiastic about their right to access, however only a minority actually consulted their medical files, with fear for confusion and anxiety being found as the main reasons for not doing so. • Some patients were disappointed in the written assessment of their pathology once they had accessed their medical records; there were privacy concerns as to who is able to access their personal ‘sensitive’ information. • In the intervention studies identified, the majority had a positive experience, generally experiencing less anxiety and feeling reassured with improved communication with their physician.
Why facilitate patient access to medical records (Ferreira et al., 2007) [11]	14 articles all focussing on patients in the outpatient setting. Predominantly US (7/14) and UK (6/14) studies.	Systematic literature search of Medline and Scopus researching the effects on medical practice of patient access to records. Thematic analysis of the results was undertaken and then each study was graded depending on how relevant they were to each theme.	**Examined the effect of patient accessibility to medical records on medical practice:** • Positive effect on the patient (promoting reassurance and reducing anxiety) and the perception of the patient-doctor relationship (breaking down barriers). • In practice, patients identified and corrected mistakes in their medical records in general practice. There were mixed results regarding adherence, and the system required familiarity with the Internet which may disadvantage some users.

Real-time access to medical records (particularly as they are currently written) may have unintended consequences on patient care both directly and indirectly – for example, by altering how things are recorded in the notes.

In this paper, we focus on adult access to notes in the medical acute care setting. We define this as the environment which comprises an adult medical patient’s presentation to hospital and their initial (up to 5 days) in-patient stay. This is a busy environment in which a sick patient generally only receives verbal communication, and in which decisions need to be made quickly, often by or with clinicians unfamiliar to the patient. In this context, access to notes may serve a different purpose than in the chronic disease or outpatient setting. Our aim was to review empirical papers relating to patient access and contribution to medical records, and consider the ethical issues raised by this proposed change in practice to fully appreciate the consequences of access to notes in real time.

Our review therefore set out to answer two questions:

1) What studies have there been of sharing records with medical patients in the in-patient setting, and in particular on the impact on trust and communication between patients and doctors?

2) What are the ethical issues associated with sharing records with medical patients?

## Methods

This study employed a systematic review framework in two stems, to integrate literature identified from two searches surrounding our research questions. This ensured a robust, replicable searching strategy from which we could extract data clearly defined by inclusion and exclusion criteria (an initial attempt to search ethical issues relating to sharing medical records in acute care yielded no relevant results). We conducted critical interpretive synthesis [16] to the data extracted, an application of qualitative enquiry that allowed us to critically analyse and integrate both the qualitative and quantitative results of both searches into main themes.

### Protocol

The review was registered on the PROSPERO database (registration ID CRD42018114125). PRISMA guidelines have been used to inform the methodology and write up.

### Identification of studies

A replicable search strategy was developed to answer our two research questions, using two literature searches, on the Medline via OVID, CINAHL via Ebsco and Scopus databases (See Appendix 1 for the full search strategies for both searches). Searches were run on the 23rd February 2018. Reference lists of included studies were reviewed for additional papers. A complete record of all identified articles was kept on a managed reference database.

#### Literature search of the empirical data

Search words, phrases and subject headings (including MeSH) were used to search for literature surrounding the topics of (1) hospitalised patients, (2) patient access to records and (3) its effects on communication and trust within the doctor-patient relationship.

The inclusion criteria limited the literature to studies about adult, hospitalised patients in the acute setting. Limits were applied for English language papers published since 1997 were included. Exclusion criteria consisted of paediatric, disease-specific studies and those focussed on confidentiality and data sharing. Studies relating to the design of a system allowing patient access to records were also excluded.

#### Literature search of the ethical issues

The second search consisted of a range of terms for (1) patient access to records and (2) ethical implications. This search therefore did not specify hospitalised patients or the effect access to notes has on communication and trust and was run from inception to the search date. The exclusion criteria remained the same.

### Study selection

**Fig. 1:**
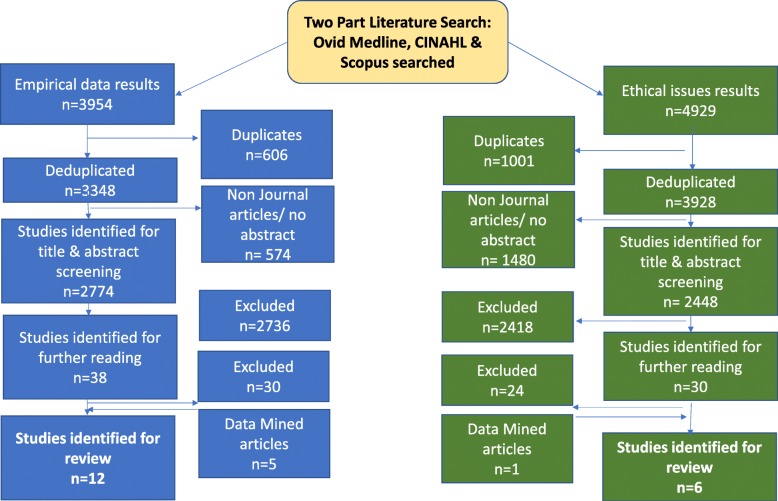
PRISMA Article Selection Flow chart

For each search, the titles and abstracts of references were screened by one reviewer (either SD or ZF) who selected those appropriate for full text analysis. 100 references in every 1000 were independently screened by both reviewers to assess for concordance and prevent drift, refining the inclusion criteria if needed. Any references where there was ambiguity were discussed by both authors and a decision made. Reference lists of included studies were screened by both authors. The results of the study selection are shown in Fig. 1.

### Data extraction and risk of bias

SD extracted the following data from the included studies: setting, nature of study, sample size, nature and contribution of participants, nature of analysis and summary of results, shown in Table 1. Both researchers conducted thematic analysis on the papers, identifying four major themes.

### Planned methods of analysis

Meta-analysis was inappropriate for the heterogenous nature of the search results and therefore a critical interpretive synthesis [16] was undertaken to discover emerging themes from the literature. Analysis of the papers was followed by extraction of data and discussion between the two authors, to consider the themes underlying these results. The ethics literature, which encompassed a wider range of settings than the empirical literature, was examined for themes which would be applicable across health care settings. An iterative process was utilised, examining and grouping them into overarching themes that both organised and illustrated the findings of the review.

## Results

Of the 3954 empirical and 4929 ethical studies identified through the two searches, 18 papers representing 16 studies were identified for review (12 empirical and 6 ethical) see Fig. 1.

Two studies used questionnaires [17, 18]; four used interviews or focus groups [19–24]; two used mixed methods [25, 26]. One note analysis, [27] one portal analysis [28] and one clinical trial [29, 30] was conducted, and six analysis articles were identified [31–36]. One empirical study came from each of Israel [19], Norway [20, 21] and Canada [18]; the rest originated from the USA. No papers looked at perspectives of the multidisciplinary team. The data extraction is summarised in Table 2.

**Table 2:** Summary of literature included. A summary of the data extracted from the included studies, themed around nature of study and research question answered.

	Author and Title of paper	Setting	Nature of Study (including whether participants are reporting on experiential or hypothetical views)	Sample Size	Nature of Analysis	Summary of results	Limitations
Questionnaires	Giving Doctors’ Daily Progress Notes to Hospitalized Patients and Families to Improve Patient Experience (Weinert, 2017) [17]	University hospital	Questionnaire of patient and healthcare providers’ response to a trial giving patients a daily copy of ‘progress notes’ (Experiential)	Patient, family members and providers (attendings, residents or medical students) involved. Pilot study: 12 patients, 6 providers, 70 notes 2nd study: 73 patients, 6 providers, 677 notes given of 2011 notes printed, (33.7%)	Quantitative analysis of 2 survey results (one for patients and family members, one for providers)	Most patients (76%) responded favourably to reading notes as it improved their understanding and feeling of control. Some providers (9–28%) thought it affected their practice: they were more careful about phrasing things and it lengthened the consultation mildly. The majority of providers (up to 72%) disagreed with the idea that it affected practice and 3–16% were neutral. There were occasions where what was written caused discord between patient and doctor, ultimately leading to better understanding: a patient with severe pain complained she did not have ‘mild pancreatitis’ and the doctor explained it was biochemically mild.	The sample size and duration of this study limited its external validity.
	Is Canada ready for patient accessible electronic health records? A national scan. (Urowitz et al., 2008) [18]	Emails to CEOs of general and acute medical hospitals in Canada	Questionnaires given to CEOs to measure national readiness for adoption and implementation of EHRs. In addition, the CEOs were asked to forward the questionnaire onto chiefs of medicine, nursing and informatics staff or other relevant people. (Experiential)	No patients were involved. 83 CEOs surveyed. Only 3% of these CEOs had assessed staff perceptions directly.	Statistical analysis and descriptive statistics.	Interview participants included 9.4% CEOs, 3.8% Chiefs of Medicine, 11.3% Chiefs of Nursing, 7.6% Chief Information Officers and 67.9% listed as ‘Other’ (the majority of which had more administrative roles: managers, privacy officers, etc). 54.2% of hospitals had some sort of EHR. Barriers to patient access were identified as hospital finances, patient computer literacy and clinician buy-in. Staff perceptions (data from 3 hospitals only): Less than 25% thought patients want access to EHR and only 16% thought they’d want lab results, in contrast with other Canadian studies that say patients/public would like access. Only 3.6% said staff would be willing and eager to provide access, 28.6% said staff would be hesitant but willing to provide access, 17.9% thought staff would support partial access.	Medical staff were surveyed minimally in this study, with the emphasis on those in more managerial roles. The response rate may be inaccurate as the distribution of the questionnaire by each CEO was not tracked.
Interviews & Focus groups	Building and testing a patient-centric electronic bedside communication center (Dykes et al., 2013) [22]	Acute care in 2 academic medical centres, USA	Focus groups to identify improvements for the electronic prototype. Pilot testing electronic bedside communication prototype including scheduled events, daily routine and space to write notes. (Experiential, real time access)	No healthcare practitioners were involved. Focus groups included former patients, family caregivers and hospital volunteers who had recently been inpatients or caregivers Pilot testing and interviews were done by 11 participants (8 inpatients and 3 family members) .	Mixed methods: Focus groups and bedside interviews	The majority of patients said they would use the device. An 82 year old said she does not want ‘one more thing to worry about’ but her family said they would use it. A 90-year-old patient said he would prefer to speak to humans directly. Accessibility issues were noted: most patients older than 64 had trouble with the touch-screen hardware. Recommendations included videoconferencing tools and voice recognition.	The method of the analysis of the interview and focus group results is unknown. The pilot testing is also limited by its small sample size.
	Designing Patient-Centric Information Displays for Hospitals.” Proceedings of the SIGCHI Conference on Human Factors in Computing Systems (Wilcox et al., 2010) [24]	Emergency Department, USA	Trial of implementing in-room displays based on medical records. (Experiential, real time access) The design of these displays involved comments and observations from collaborating physicians.	18 patients, 11 visitors (all female), 16 physicians. Patients were interviewed after deploying the prototype, then again when all further medical updates had been made to their prototype.	Semi structured interviews with patients, family and healthcare practitioners.	17 patients overwhelmingly positive about the poster display (which included a health profile, vitals, what’s next, medications, care team) because it helped them keep track, especially the ‘What’s Next’ section. One participant said: “yeah, they come in and update me but, I mean, I can’t keep track of it all. That’s why I really like this.” Physicians were also positive but expressed concerns over display of lab results and vitals.	The study gives limited detail about the structure and method of analysis of each interview, particularly for visitors and physicians. Patients were recruited for the study by collaborating physicians, which may have introduced selection bias.
	Why do people want a paper copy of their electronic patient record (Wibe et al., 2010) [20]	2 hospitals in Norway Three papers from one study.	Interviews with Norwegian adult patients who have requested access to their notes (Experiential, retrospective access)	A convenience sample of 17 volunteers following an inpatient stay: 16 female, 1 male. No physicians or other healthcare practitioners were interviewed.	Qualitative content thematic analysis	A main priority for patients is the secure transmission of information between healthcare personnel; they want to take it upon themselves to be the ‘messenger’.	The population of this study represents less than 1% of those admitted in the same time frame. The retrospective nature of this study may have increased the recall bias.
	Patients reading their health records - what emotional factors are involved? (Wibe et al., 2009) [37]					Distrust is an important motivator for asking for record access, but some have more practical reasons – e.g. insurance. Discrepancies (mixing up records of 2 different patients) and lack of openness cause irritation and resentment in individual health care worker and in the system.	
	Lay people’s experiences with reading their medical record (Wibe et al., 2011) [21]					Very few patients (1%) of those admitted to hospital request their records – and this research interviewed only this selected sample. Patient reasons for requesting records include desiring a sense of control, taking responsibility and examine inaccuracies. Problems reading the record included patients feeling like they weren’t being taken seriously and the record stigmatising their lifestyle problems.	
Note Analysis	Patient-centric medical notes: Identifying areas for improvement in the age of open medical records (Lee et al., 2017) [27]	Tertiary care centre, USA	Retrospective analysis of patient notes looking at barriers to patient access (Hypothetical)	337 inpatient admission notes. No direct information from patients or healthcare practitioners contributed to the results.	Statistical analysis of characteristics of interest (e.g. offensive medical language) identified in notes.	The notes that create confusion, generate offense or impact perceptions/professionalism were those that used medical words which may have judgemental connotations in (e.g. ‘complains’, ‘claims’, ‘denies’), typographical errors and use of jargon.	This study did not look at patients directly accessing notes, but rather what within the notes creates barriers to access. These characteristics were determined by clinicians, rather than involving patients. This study was conducted at a single site which reduces its eternal validity.
Portal Evaluation	Implementation of acute care patient portals: recommendations on utility and use from six early adopters. (Grossman et al., 2017) [28]	6 hospitals in the USA.	Evaluation of implementing acute care patient portals. (Experiential, real time access)	6 hospital portals serving 1065 patients overall. Literature review of 27 studies looking at the characteristics of acute care patient portals.	Analysis of characteristics, usage and tools of each portal. Literature review of other studies on patient portals.	The purpose of most portals is to engage patients and facilitate transition to outpatient management, clinician relationship, transparency of information and patient safety. They take a patient centric approach with a care plan and daily schedule, and tools to facilitate messaging and accessing results; half showed the diagnosis. Problems with portals were also identified, including timing of lab result release and the sharing of differential diagnoses. There was a concern that portals would lead to ‘overwhelming amounts’ of patient contact needs.	The study focuses of large academic medical centres meaning the results may not be valid across smaller hospitals. Not all the portals focussed on communicating medical advice; some were used for non- medical information.
Clinical Trials	The effect of tablet computers with a mobile patient portal application on hospitalized patients’ knowledge and activation. (O’Leary et al., 2016) [29]	Controlled trail in a large teaching hospital	Patient portal with personal health information (e.g. names and pictures of team members, scheduled tests, and list of active medications) presented on 15 iPads, given to 100 intervention patients. (Experiential, real time access)	100 intervention patients, 102 control patients. Each physician in charge of the care of each patient was also interviewed.	Structured and semi-structured interviews with patients and their physicians separately – responses recorded verbatim and compared to each-other and the medical record. Short form measuring Patient Activation (PAM-SF) qualitatively.	A larger percentage of intervention patients (56%) named 1 or more physicians compared to the control group (29.4%). Similarly, more intervention patients (47%) could name the role of one or more physicians compared to the control group (15.7%). There was no difference in patient groups knowledge of all their planned tests or procedures. Patient activation (the level of knowledge, skills and confidence a patient has in managing their own healthcare) from the PAM-SF score remained unchanged between the groups. Of those who had access to the portal, 57% used the portal more than once a day. The majority of patients thought it was useful and easy to use. Reasons for lack of patient activation could include terminology interpretation and lack of time. Further improvements include working to engage patients more and make portals more accessible.	Study did not assess health literacy of their patients and was limited to English-speaking patients. The study was conducted on one site only.
Mixed Methods	A tablet computer application for patients to participate in their hospital care. (Vawdrey et al., 2011) [25]	Cardiac Step-down unit, USA	Mixed methods: semi-structured interviews and questionnaires. (Experiential, real time access)	5 patients interviewed (all male) Clinician reception of the pilot study was noted although not assessed formally.	Analysis using subscales for satisfaction and usefulness within the questionnaire, and anecdotal evidence from interviews.	Patients found portal helped them engage more with their care and form a more personal relationship with the MDT. None of the patients raised privacy concerns.	Small population of study on one specialised unit, so results may not be consistent across all acute care settings.
	Acute care patient portals: a qualitative study of stakeholder perspectives on current practices. (Collins et al., 2017) [26]	Acute care in 5 academic medical centres, USA	Mixed methods: semi-structured interviews, focus groups, site visits and questionnaires of expert leaders at each site to evaluate perceptions of patient portals and identify requirements of patient portals in the acute care setting. (Hypothetical)	84 participants in total including on average: 3.3 PFAC (patient family advisory council) members 4.5 researchers 3.8 Information system leaders 3.6 clinical leaders 1.3 policy makers and administrators	Thematic analysis of 12 interviews and 18 focus groups to form development of an explanatory model.	Main themes identified from stakeholders include: access and security (with concern over BYOD use outside of hospital); content and functionality encouraging simplistic intuitive displays; the need to minimise the exclusion of those with less health literacy and engagement; using both patients and doctors in the design and training to use the portal. Patients believed the portal would facilitate face-to-face communication rather than replace it. The portal must be easy to use and ‘familiar’; this was considered particularly important for the acutely-unwell patient.	The study was limited to academic centres and as such, results may not be valid across all acute care settings.
	Research question 2: What are the ethical issues associated with patients having access to their medical records?						
	The challenges in making electronic health records accessible to patients. (Beard et al., 2012) [31]	–	Examining concerns arising in relation to patient access to health records. (Hypothetical)	–	Analysis	1.Cost and security concerns: limited financial resources need to be shared across healthcare organisations and clear regulations regarding access must be communicated. 2.Assignment of responsibilities and rights: there are conflicts regarding the timing of information (release of lab results); use of medical terminology and control of the record. 3.Liability issues:the use of messaging portals may present a liability risk. 4. Tensions between patients and doctors: is messaging appropriate; are physicians proficient with electronic communication.	
	Ethical Considerations about EHR-Mediated Results Disclosure and Pathology Information Presented via Patient Portals. (Davis and Smith, 2016) [32]	–	Examining ethical issues regarding patient access to pathology and other results (Hypothetical)	–	Analysis	The main focus was on time delays for different pathology reports to be accessible to patients. Some results (like HbA1C) are useful for patients to monitor the progress and for peace of mind, improving self-reliance but patients may feel to blame for poor outcomes due to lack of vigilance. Having access to other results could have negative effects: Access to abnormal, ‘surprising’ results could lead to patients having to come to terms with a diagnosis without emotional support or the immediate opportunity to ask questions; the (lack of) importance of out-of-range results could be misunderstood; genetic testing results and diagnoses might be seen without sufficient counselling.	
	Legal, Practical, and Ethical Considerations for Making Online Patient Portals Accessible for All. (Lyles et al., 2017) [33]	America	Examination of the legal, practical and ethical issues regarding patient portals in America. (Hypothetical)	–	Analysis	Interest in portals is constant throughout populations but portals themselves are mostly small-font, English only, text-based content and therefore disadvantage disabled users and non-English speakers. There are no specific regulations regarding the accessibility standards of EHRs, but portal designs must take the Civil Rights act and the Digital Accessibility Guidance into account.	
	Why a shared care record is an official medical record. (Gu et al., 2013) [34]	New Zealand	An argument supporting the validity of a shared care record as an official record and the consequences of this. (Hypothetical)	–	Analysis	A shared record needs to meet ethical and medico-legal criteria, including regulations for interoperability, clinical responsibility and restrictions on patient and professional access. Issues include balancing empowerment with legislative requirements and how we can foster confidence in healthcare professionals to co-partner the record.	
	Ethical questions must be considered for electronic health records. (Spriggs et al., 2012) [35]	Australia	Identifying concerns over electronic health records (Hypothetical)	–	Analysis	This study identified specific questions arising from the move towards personally controlled electronic health records in Australia, including who benefits and who should pay for the system, what uses of the system are legitimate, how we should govern the management and use of the system and how we should implement privacy. Three main questions were identified for our analysis: 1.Can records be considered trustworthy if patients can lock/change record? 2.Will patients who do not (or can not, for health literacy reasons) use the system be disadvantaged? 3. Who else can access the information? Consumers generally more willing to share information for research and public health uses but not for pharmaceutical use.	
	Medical records: practicalities and principles of patient possession. (Gilhooly and McGhee, 1991) [36]	UK	Examining the practical and ethical advantages and disadvantages of patient possession of medical records. (Hypothetical)	–	Analysis	Practical benefits include storage and transfer of notes – this would be less problematic if the patient possessed them and the patient can check/screen notes regularly. Practical problems include the risk of patients losing records (although research shows otherwise), the extra time doctors may take to explain the record’s contents (although the benefit of the patient understanding may outweigh the cost of time lost). Ethical benefits include reducing the power imbalance between the doctor and patient, leading to more communication and trust as patients can control confidentiality of records. Ethical problems include that the doctors may feel they need to censor record so as to not offend patients. There is an argument that the notes are doctors’ property and that patients might feel anxious due to the medical uncertainty portrayed through the notes.	

Four main themes emerged on analysis: Impact on patient care; Conflicts between patient and physician perspective; divergent views on doctor and patient roles; cultural differences and societal risks.

### Impact on patient care

Sharing notes was seen to empower patients by improving trust and knowledge [30], facilitating patients to work with doctors [28]. Communication of written information was considered superior to verbal explanations; one patient was reported as saying *“Yeah, they come and update me but..I mean I can’t keep track of it all. That’s why I like this.”* [24] No studies revealed objective changes in care such as reduced length of stay. Access to their own notes might enable patients to correct inaccuracies, [21, 36] although this raised the possibility of patients feeling responsible if something was missed: [20, 32]

*“patients could end up feeling they are to blame for their own poor outcomes.”* [32]

Some participants thought written information might ‘facilitate verbal communication’. [26] Others were concerned that a written note might supplant face-to-face interaction [22]; this did not manifest in the only study to trial giving patients a written daily summary [17].

### Conflict between doctor and patient perspectives

Patients and doctors had discordant perceptions of how accessing the medical record might affect care: whilst doctors were concerned access to notes will overwhelm or unnecessarily worry patients, [17, 24] patients were reassured by the shared information [37]. Grossman et al suggested that *‘it may be prudent to omit or explain potentially alarming information that carries a low degree of certainty such as a cancer on a differential diagnosis list”* [28]*.*

A reoccurring conflict was the release of lab (and other) results in real time – patients strongly supported this whereas doctors preferred a delay, [24, 29, 31] in part so they could interpret them appropriately, offer support and create a future healthcare plan. Without this, some participants theorised that results could be prone to misinterpretation and unnecessary anxiety could be provoked [32]. As a physician participant said: *“one of the primary duties of a physician is not only to alert the patient to abnormal results but also to educate them on their condition and appraise them of the follow up that will be needed”* [32]*.* If delayed release did exist, however, there was a question about who would take responsibility for this [31].. Interestingly, this was not mentioned in the papers reporting direct experience.

There was also debate about whether patients should be co-creators of notes: Doctors, again hypothesising, were concerned that patients editing their own record might make them less reliable [34, 35].

### Divergent views on doctor and patient roles

A range of alternative approaches have been developed to share non-verbal information, and they reveal a variety of implicit perspectives about the role of the patient and the doctor. Tools designed to ensure patient choice and satisfaction are for those who perceive the patient as *client*; one participant was quoted as saying: “I would like to be able to see background information [ about my doctor] like where they went to school” [25]. Providing information in the hope that patients will become more actively involved in their care see the patient as *collaborator* [22, 29]. The different perspectives influence the purpose (and extent) of information sharing.

### Cultural difference and societal risks

Different healthcare systems worldwide vary in their approach and concerns regarding access to notes – one study set in Israel found that the doctors more willing to share notes with patients originated from English-speaking countries, suggesting a cultural influence towards this [19]. In some countries such as the USA and Norway, liability seems to be more of a concern for the doctors and more of a motive for patients to access notes [21, 24].

Across geographical boundaries, however, there was a recognition that there would be variation in patients’ willingness and ability to access notes, and that this might lead to disparity in health care, [22, 35] with those from lower socioeconomic groups less likely to engage despite an often greater need; *‘{to] what extent should less engaged individuals be punished for their ‘ignorance’* [35]*.* As Lyles et al stated*: “there is an ethical imperative to work to reduce the potential for the emergence or amplification of health disparities with respect to portal use’* [33]*.* Large screens, simple formats and buttons will help accessibility for some [26, 38]; empirical research assessing the impact on access to health care or impact on different socioeconomic groups was not identified.

Finally, the questions of privacy and security of patient notes were raised, although papers focussing solely on this issue were excluded from the study. Some patients were concerned about the security of having information on their own devices, [26] while others did not voice privacy concerns [25]. Patients need to be able to trust their details are stored and shared securely, so they can contribute to them in a transparent manner [35].

## Discussion

The review reveals a consensus that our current approach to giving information to patients – almost exclusively verbally – is insufficient; that patient access to notes is a welcome next step for patient-centred care, but that simply allowing full access, without explanation or summary, is also insufficient. Several ethical implications need to be considered: increased information could improve patient trust and knowledge but might transfer an (unwelcome) sense of responsibility to patients; doctors and patients have conflicting views on how much information should be shared and when; sharing written information might increase the already significant disparity in access to health care, and have unforeseen opportunity costs.

It is also clear that we need to consider the impact that sharing notes in real time will have on medical practice.

### Trust and the medical record

Although trust, both in doctors individually and generally, is often measured, it is rarely sufficiently specified in the medical literature. Trust is necessary when there is a degree of uncertainty and vulnerability (Becker 1996), both of which are present in the patient-doctor relationship; uncertainty about diagnosis and treatment, vulnerability not only because the patient is physically unwell, but because of the anxiety which often accompanies illness, and which can affect judgment. Trust is often described as a ‘three place relation’: ‘A’ trusts ‘B’ with ‘C’ [39].

In healthcare, the factors which can determine trust can relate to the patient (‘A’) and the doctor (‘B’), as well as what is entrusted (‘C’), namely the patient’s care [40]. Since the degree of care required is related to the severity and circumstances of the illness, these are also factors which can affect the patient’s vulnerability and need to trust. While trust is necessary for a functioning patient-doctor relationship, too much trust could be detrimental [41]. It may lead to reduced patient involvement in decision-making, or fewer questions being asked, leading to the possibility of sub-standard patient care.

What we want to achieve is well-placed patient trust, a concept O’Neil refers to as trust of the trustworthy, [42] where a patient can be confident that their trust in their clinician is justified and thus can reasonably entrust decisions and actions about their care to him or her. This places an obligation on clinicians to be trustworthy, but it also requires patients to be able to ask questions to satisfy themselves that their trust is well-placed. Providing access to medical records enables patients to determine what they are entrusting (more about what is wrong with them, and more about what treatments and investigations are planned) and enables them to place their trust well (or withhold it). Patients reading their own records might in turn alter physicians’ behaviours to be more trustworthy: they may, as they have done with clinic letters, modulate their language and ensure better verbal communication to avoid misconstruction of what is written.

### Increased knowledge, increased responsibility?

While trust is important, the relationship between trust and autonomy has been well explored [43]. In medical ethics analysis of the last 40 years, autonomy has been given primacy [44, 45]; part of respecting patient autonomy is ensuring that they have sufficient information to participate in shared decision-making [46]. There appears to be a recognition that the current approach – of only relaying verbal information to patients until their discharge – is inadequate. Patients forget, [47] relatives are concerned, questions are not asked [48].

It is thus unsurprising that imparting more (or more accessible) information to patients was welcomed by both patients and doctors. However, concerns were expressed that giving more information to patients also transferred responsibility to them: responsibility to check for errors; to deal with uncertainty; to worry about results. This responsibility may not always be desired by the patient. As Alfred Taubert says: “In the so-called co-operative mode, guidance dominates to the point where most patients, realistically and appropriately, want the doctor to take responsibility for their health.” By giving patients increased information, we may be removing their choice to defer responsibility – and associated ‘emotional work’ [49] or worry - to their physician.

### Too much information, too soon?

A specific example of emotional work or worry related to receiving test results in real time: whilst patients expressed a strong desire for this, doctors’ concerns are two-fold. Firstly, they were concerned that patients lack the medical expertise to gauge the clinical importance of results. Secondly, they were worried that they (the doctor) would not be present to offer support and interpretation if the patient receives distressing news. Receiving emotional support from their doctor was a primary reason found for why patients audio-record consultations [50]; getting results without the doctor present would deprive them of that immediate support. Outside the acute setting, Milliat-Guittard showed that 21% of breast cancer patients did not want to hold records; they did not want to come across a comment that they were not expecting. Instead, they wished to come to terms with the disease in their own way [51].

### Unintended worsening of inequality

Some interventions unintentionally increase inequalities by disproportionately benefiting less disadvantaged groups [52]. Giving patients access to records might be one such intervention: clinical teams acknowledged that they were working in a stretched system - an intervention which could divert resources to those who could read and understand their medical notes (or who had the confidence to ask questions) might lead to disparities. Awareness of this, and establishing and testing ways to mitigate this risk would be an important element to consider when introducing shared medical records.

### Impact on medical practice

Medical records are not only a patient narrative – of their presentation, their investigations and their progress - but a working medical document which reflects dynamic thinking, [53] consultations, and acts as a tool for handover and for training [54]. If doctors do not reflect concerns clearly in the notes for fear of worrying the patient, handover could be compromised, impacting negatively on the patient’s care and training of future doctors.

### Strengths and limitations

This review synthesised a wide range of papers from medical, nursing and ethical literatures, and was rigorously conducted. However, it identified only papers written in western cultures, and in English, and the conclusions made here should not be extrapolated to other environments. In addition, 7/10 of the studies were carried out in the USA, where the patient doctor relationship also includes a transactional component – doctors need to ensure that patients know what they are paying for. In other health systems represented in these studies (Canada, Norway, Israel) this is not the case, and so the motivations and repercussions of information sharing may be different.

### Conclusions and future directions

These studies - and the timing of their publication - reveal that there is significant growth in the approach of sharing more medical information with patients, and significant variation in the type and quantity of information which is being shared. Empirical work with integrated ethical analysis is needed examining the impact of sharing medical records on patient-doctor and multi-disciplinary team communication, on patient trust, on physician training and on resources. The overarching question is what changes will occur to the role of doctor and patient as a result of routinely sharing more information, and, normatively, if there is a “right” amount of information to share with patients in the hospital setting.

Sharing information is a critical part of clinical practice; changing how it is done could have significant empirical and ethical impacts. This review has highlighted what those potential impacts might be. We recommend that careful evaluation of what is recorded and what care is given – both at individual and societal levels – need to be conducted when changes are made to how information is shared.

## Supplementary information

**Additional file 1.** Full search strategies for both literature searches.

## Data Availability

There are no further data to present other than that which is presented here.

## Abbreviations

CINAHL: Cumulative index of nursing and allied health literature
EBSCO: Elton B Stephens Company (A database search database)
MeSH: Medical subject headings
UK: United Kingdom
USA: United States of America
