# Supplementary material for: A systematic review of patient access to medical records in the acute setting: practicalities, perspectives and ethical consequences
Source: BMC Med Ethics. 2020 Mar 2;21:18. doi: 10.1186/s12910-020-0459-6 (PMC7053049; doi:10.1186/s12910-020-0459-6)
Supplement: Supplementary file 1 — Additional file 1. Full search strategies for both literature searches. [file 12910_2020_459_MOESM1_ESM.docx]

| **Author and Title of paper** | **Sample Size and setting** | **Nature of Analysis** | **Summary of results** |
| --- | --- | --- | --- |
| **Electronic Patient Portals:** Evidence on Health Outcomes, Satisfaction, Efficiency and Attitudes.  (Goldzweig et al, 2013) | 46 included articles: 18 on health outcomes, 7 on efficiency/utilization, 10 on patient characteristics, 19 on attitudes  All outpatient setting , assessing portals as a way of accessing information. Predominantly US (43/46) | 3 systematic literature searches of PubMed and Web of Science spanning different timeframes for the **effects of portals on patient care**. Included reference -mined articles Assessed independently by two reviewers. | **Examined health outcomes for specific diseases** (e.g. effect of intervention on HbA1C levels in diabetics) rather than patient communication and dr-patient relationship.  **Health outcomes, satisfaction and adherence:** positive or neutral outcomes in intervention patients compared to control patients in RCTs for patient portals for those with chronic conditions; possible confounding factors, as portals used in conjunction with intensive or pharamaceutical-led case management.  **Efficiency or Utilization:** either no difference or increased clinician contact  **Patient characteristics:** disparity between racial and ethnic groups, literacy or education levels and medical problems in regards to whether the patients were likely to use portals or not, suggesting this is a barrier to accessible portal use. |
| Inpatient Portals for Hospitalized Patients and Caregivers: A Systematic Review (Kelly et al, 2017) | 17 studies | Systematic literature search of PubMed, Web of Science, CINAL Plus, Cochrane and Scopus for **patient portals, engagement and inpatient care.** | **Examined the design, use and impact of patient portals.**  Portals provided targeted access to information for patients, and were varied in their design and content.  Patients generally found portals easy to use and have a positive experience, feeling more engaged and in control; they suggested future portals should have more information, personalised medication and results in real time.  Professional concern over giving information without interpretation and overuse of messaging tools. |

| Patient engagement in the inpatient setting: a systematic review.(Prey et al, 2014) | 17 studies | Systematic literature search of PubMed, ACM Digital Library, IEEE Xplore and Cochrane databases for **patient engagement, involvement of health I.T. in an inpatient setting** (English language only) | **Examined what Interventions to improve patient access engagement via health information technology were available.**  Five groups were identified: entertainment, general health I.T. delivery, patient-specific information delivery, advanced communication tools and personalized decision report.  Noted limited research on impact on health outcomes and cost-effectiveness. |
| --- | --- | --- | --- |
| The effects of promoting patient access to medical records: a review (Ross & Lin, **2003)** | 41 included articles including medical outpatients, inpatients, obstetric, and psychiatric patients | Systematic literature search of MEDLINE and HealthSTAR searching for the effects of patient access to notes on patient participation and advocacy. Reference mining was also used. | **Examined effects of patient access to records over a period of time on the patient, the doctor-patient relationship and medical practice.**  Patient access to medical records was unlikely to cause harm and generally had modest benefits, especially surrounding doctor-patient communication, seen clearly in three trials in obstetric intervention patients.  Patient satisfaction was generally high, despite some patients finding the records worrisome, upsetting or difficult to understand. |
| The patient perspective on the effects of medical record accessibility: a systematic review (Vermeir et al, 2017) | 12 studies | Systematic literature search of PubMed, Web of Science, Cinahl and Cochrane Library for effects of communicating medical record information on patient participation and the doctor-patient relationship. The studies were assessed for methodological quality and those scoring average and high ratings were included. | **Examined patient use of and perspectives on medical record accessibility**  Many patient participants were knowledgeable and enthusiastic about their right to access, however only a minority actually consulted their medical files, with fear for confusion and anxiety being found as the main reasons for not doing so.  Some patients were disappointed in the written assessment of their pathology once they had accessed their medical records; there were privacy concerns as to who is able to access their personal ‘sensitive’ information.  In the intervention studies identified, the majority had a positive experience, generally experiencing less anxiety and feeling reassured with improved communication with their physician. |
| Why facilitate patient access to medical records (Ferreira et al, 2007) | 14 articles | Systematic literature search of Medline and Scopus researching the effects on medical practice of patient access to records. Thematic analysis of the results was undertaken and then each study was graded depending on how relevant they were to each theme. | **Examined the effect of patient accessibility to medical records on medical practice:**  Positive effect on the patient (promoting reassurance and reducing anxiety) and the perception of the patient-doctor relationship (breaking down barriers).  In practice , patients identified and corrected mistakes in their medical records in general practice. There were mixed results regarding adherence, and the system required familiarity with the Internet which may disadvantage some users. |
